# Decorin: a potential therapeutic candidate for ligamentum flavum hypertrophy by antagonizing TGF-β1

**DOI:** 10.1038/s12276-023-01023-y

**Published:** 2023-07-03

**Authors:** Shanxi Wang, Yunkun Qu, Xuan Fang, Qing Ding, Hongqi Zhao, Xiaojun Yu, Tao Xu, Rui Lu, Shaoze Jing, Chaoxu Liu, Hua Wu, Yang Liu

**Affiliations:** 1 Department of Orthopedics, Tongji Hospital, Tongji Medical College, Huazhong University of Science and Technology, Wuhan, People’s Republic of China; 2 Department of Orthopedics, Third Hospital of Shanxi Medical University, Shanxi Bethune Hospital, Shanxi Academy of Medical Sciences, Tongji Shanxi Hospital, Taiyuan, People’s Republic of China

**Keywords:** Neuromuscular disease, Transdifferentiation, Drug development, Ligaments, Molecularly targeted therapy

## Abstract

Ligamentum flavum hypertrophy (LFH) is the main physiological and pathological mechanism of lumbar spinal canal stenosis (LSCS). The specific mechanism for LFH has not been completely clarified. In this study, bioinformatic analysis, human ligamentum flavum (LF) tissues collection and analysis, and in vitro and in vivo experiments were conducted to explore the effect of decorin (DCN) on LFH pathogenesis. Here, we found that TGF-β1, collagen I, collagen III, α-SMA and fibronectin were significantly upregulated in hypertrophic LF samples. The DCN protein expression in hypertrophic LF samples was higher than that in non-LFH samples, but the difference was not significant. DCN inhibited the expression of TGF-β1-induced fibrosis-associated proteins in human LF cells, including collagen I, collagen III, α-SMA, and fibronectin. ELISAs showed that TGF-β1 can upregulate PINP and PIIINP in the cell supernatant, and this effect was inhibited after DCN administration. Mechanistic studies revealed that DCN suppressed TGF-β1-induced fibrosis by blocking the TGF-β1/SMAD3 signaling pathway. In addition, DCN ameliorated mechanical stress-induced LFH in vivo. In summary, our findings indicated that DCN ameliorated mechanical stress-induced LFH by antagonizing the TGF-β1/SMAD3 signaling pathway in vitro and in vivo. These findings imply that DCN is a potential therapeutic candidate for ligamentum flavum hypertrophy.

## Spinal health: Keeping a key ligament elastic

A protein called decorin offers a potential therapy for ligamentum flavum hypertrophy (LFH), stiffening of the ligament inside the spinal canal that connects vertebrae. In LFH, fibrosis (deposition of extra collagen) thickens and stiffens the ligament, reducing the space in the spinal column, thereby compressing nerves and causing loss of mobility. Yang Liu and Hua Wu at Huazhong University of Science and Technology in Wuhan, China, investigated whether decorin, known to combat fibrosis in other contexts, could help treat LFH. Treating LF cells with decorin decreased levels of proteins associated with fibrosis. Testing in a rodent model of LFH showed that treatment with decorin reduced ligament thickness and the amount of collagen. Further testing indicated that decorin suppresses signaling that leads more collagen to be laid down. These results suggest a potential treatment approach for LFH.

## Introduction

Lumbar spinal canal stenosis (LSCS) is one of the most common causes of lower back pain and gait disorder in elderly individuals and seriously affects the daily living activities and health of patients^1^. Disc herniation and ligamentum flavum hypertrophy (LFH) are the main physiological and pathological mechanisms of LSCS, which can compress the dura, cauda equina, and nerve roots, resulting in corresponding clinical symptoms^2–4^.

The ligamentum flavum (LF) is an important part of the posterior spinal column. Its main function is to limit the hyperflexion of the spine and play a role in maintaining the stability of the spine together with the intervertebral disc, facet joint and intervertebral ligament. In addition, the LF is an important part of the posterior lateral wall of the spinal canal and has a protective effect on the spinal cord^5^. Histologically, normal LF is composed of 80% elastic fibers and 20% collagen fibers^6^. However, LFH manifests as the degradation of elastic fibers and an increase in collagen fibers, which are typical fibrotic changes^7^. Hypertrophic LF can compress nerves or the spinal cord, causing clinical symptoms such as numbness, pain, and limited mobility^8–10^.

Many studies have shown that fibrosis is the main cause of hypertrophy of the LF, and a variety of molecules participate in this pathological process, including transforming growth factor beta 1 (TGF-β1), connective tissue growth factor (CTGF), wnt-induced secreted protein-1 (WISP-1), interleukin- 1β (IL-1β), interleukin-6 (IL-6), matrix metalloproteinases (MMPs), fibroblast growth factor (FGF), vascular endothelial cell growth factor (VEGF), etc.^4,7,11–14^. Among them, TGF-β1 is considered to be one of the factors most closely related to LFH^11,15–18^.

Decorin (DCN) is a small leucine-rich proteoglycan that interacts with various extracellular matrix proteins, cell surface receptors, and cell growth factors^19^. More importantly, DCN can inhibit the function of TGF-β1 by binding to it and neutralizing some of its activity and is thought to be a natural inhibitor of TGF-β1^20,21^. Many studies have shown that this molecule can improve the pathological changes caused by fibrosis in various tissues by inhibiting TGF-β1^22–27^. In the present study, bioinformatic analysis, human LF tissues collection and analysis, as well as in vitro and in vivo experiments were conducted to explore the effect of DCN on LFH pathogenesis.

## Materials and methods

### Bioinformatic analysis

Raw data were acquired from the GEO database (https://www.ncbi.nlm.nih.gov/geo). GSE113212 includes eight LF samples, four of which are from healthy people, while the others are from patients with LFH. QC (quality control) and the identification of DEGs (differentially expressed genes) were performed with the R package limma^28^. Clusterprofiler was applied to perform GO and KEGG enrichment analyses of the DEGs^29^. The R package ggplopt2 was used to draw the pictures. The STRING database was used to identify potential protein‒protein interaction (PPI) relationships (https://cn.string-db.org/). MCODE and Cytoscape (V 3.8.2) were further used to select the subnetwork.

### Human LF sample collection

Patients with lumbar degenerative diseases who needed surgical intervention were enrolled in this study. According to the preoperative diagnosis, patients were divided into the LFH group and the non-LFH group. Patients with LSCS caused by LFH were assigned to the LFH group, and patients with simple lumbar disc herniation were assigned to the non-LFH group. LFH was defined as a LF thickness greater than 4 mm on magnetic resonance imaging (MRI) at the operated level, and non-LFH was defined as a LF thickness less than 3 mm on MRI at the operated level. LF tissues (L4/5) were isolated and collected during the operation and then used for subsequent experiments.

### Cell isolation and culture

Human LF cells were isolated from LF tissues as described previously^13^. After the LF tissues were obtained from patients, the samples were washed three times with phosphate-buffered saline (PBS, Boster, Wuhan, China). Subsequently, the LF tissues were cut into approximately 0.5 mm^3^ granules and digested with 0.2% type I collagenase (Sigma, USA) for 1 h at 37 °C in a cell incubator. Then, 0.2% type I collagenase was removed, and the samples were washed with Dulbecco’s modified Eagle’s medium (DMEM, Gibco, USA). Finally, the specimens were incubated with DMEM containing 15% fetal bovine serum (Gibco, USA) and 1% penicillin‒streptomycin solution (Biosharp, Guangzhou, China) in a cell incubator with a 5% concentration of CO_2_ at 37 °C. LF cells within four generations were used for subsequent experiments.

### Cell viability and proliferation

LF cells were cultured in 24-well plates (2 × 10^4^ cells/well) or 96-well plates (1 × 10^4^ cells/well). After 80% confluence, the LF cells were treated with different concentrations of recombinant human DCN (143-DE, R&D Systems, Minneapolis, MN, USA) for 24 h. Subsequently, the Calcein-AM/PI Double Stain Kit (Yeasen, Shanghai, China) and Cell Counting Kit-8 (CCK-8, Boster, Wuhan, China) assays were used to test cell viability and proliferation. For calcein-AM/PI double staining, a fluorescence microscope (Evos Flauto; Life Technologies, USA) was used to gather live/dead cell images, and the live cells and dead cells were counted in six random fields of view for each well. For the CCK-8 assay, CCK-8 solution was added to a 96-well plate (10 µL/well), and the 96-well plate was incubated in the dark for 1 h at 37 °C. Finally, the absorbance at 450 nm was obtained using a spectrophotometric microplate reader (Bio-Rad, Richmond, USA).

### Cell intervention

LF cells derived from patients in the non-LFH group (normal LF cells) and LFH group (hypertrophic LF cells) were cultured in 24-well plates (2 × 10^4^ cells/well) or 6-well plates (1 × 10^6^ cells/well). After 80% confluence, the normal LF cells were treated with different concentrations (0 ng/ml, 2.5 ng/ml, 5 ng/ml, 10 ng/ml) of recombinant human TGF-β1 (240-B, R&D Systems, Minneapolis, MN, USA), and hypertrophic LF cells were treated with different concentrations (0 nM, 50 nM, 100 nM, 200 nM) of recombinant human DCN for 24 h. Subsequently, the cells were collected for protein extraction, and the cell supernatant was collected for ELISAs. For further analysis of the role of TGF-β1 and DCN in the LFH process, normal LF cells were treated with 10 ng/ml TGF-β1 in combination with different concentrations of DCN for 24 h. Similarly, the cells were then collected for immunofluorescence staining and protein analysis, and the cell supernatant obtained was used for ELISAs.

### Western blotting analysis

LF tissues were cut into approximately 0.5 mm^3^ granules and then homogenized with a tissue homogenizer until there were no visible solids. LF cells were collected and lysed with RIPA lysis buffer containing 1% phosphatase inhibitors and 1% protease inhibitors (Boster, Wuhan, China) on ice for 30 min. Subsequently, an ultrasonic disruptor was used for further lysis. After centrifugation, the supernatant was collected, and the protein concentration was detected with a BCA assay kit (Boster, Wuhan, China). After this, the proteins were mixed with protein loading buffer at a ratio of 4:1, heated at 100 °C for 5 min, and finally stored at −80 °C for Western blotting analysis. Proteins were separated by electrophoresis using SDS‒PAGE gels and then transferred to PVDF membranes (Millipore, Billerica, USA). The PVDF membranes were blocked with 5% skim milk for 1 h and then incubated with species-matched primary antibodies against decorin (#29199, Signalway Antibody LLC, Maryland, USA), TGF-β1 (#41494, Signalway Antibody LLC, Maryland, USA), collagen I (14695-1-AP, Proteintech Group, Wuhan, China), collagen III (22734-1-AP, Proteintech Group, Wuhan, China), α-SMA (ab124964, Abcam, Cambridge, UK), fibronectin (ab268020, Abcam, Cambridge, UK), SMAD3 (#9253, Cell Signaling Technology, Danvers, USA), phosphorylated-SMAD3 (P-SMAD3, #9520, Cell Signaling Technology, Danvers, USA) and GAPDH (BM1623, Boster, Wuhan, China) at 4 °C overnight. Next, the membranes were washed three times with TBST and then incubated with species-matched secondary antibodies (Cell Signaling Technology, Danvers, USA) for 1 h at room temperature. Finally, the membranes were washed again with TBST, and the protein bands were developed with a Western blotting chemiluminescence kit (Thermo Pierce, MA, USA) and visualized with a Bio-Rad scanner system (CA, USA).

### Immunofluorescence

After intervention, LF cells were fixed with 4% paraformaldehyde for 10 min and permeabilized with 0.1% Triton X-100 for 15 min, and then, the cells were blocked with 5% goat serum for 1 h. After this, the LF cells were incubated with antibodies against collagen I, collagen III, α-SMA and fibronectin at 4 °C overnight. Subsequently, the cells were incubated with FITC-conjugated (green) or Cy3-conjugated (red) anti-rabbit IgG antibodies (Boster, Wuhan, China) for 1 h in the dark and then stained with DAPI (Boster, Wuhan, China) for 10 min. Finally, a fluorescence microscope (Evos Flauto; Life Technologies, USA) was used to gather immunofluorescence images.

### Animals and animal procedures

Thirty male Sprague–Dawley rats (250–300 g) were randomly divided into three groups. One group was the sham group, and the other two groups were the LFH model group. The LFH model was established as described previously^30^. After weighing, the rats were anesthetized by pentobarbital (intraperitoneal injection, 4.0 mg/100 g body weight). A dorsal longitudinal incision was adopted over the L4-5 or L5-6 spinous processes of the rats. After exposure of the spinous processes and bilateral facet joints, the adjacent muscles were detached. The spinous processes, bilateral facet joints and interspinal ligament were resected. After surgery, one of the LFH model groups was treated with paravertebral injections of DCN (200 nM, 500 µl per rat, twice per week), and another LFH model group was treated with saline in the same way. After 8 weeks, all rats were sacrificed, and the intact peripheral blood and operative segmental vertebrae were isolated and collected for further experiments.

### Enzyme-linked immunosorbent assay

LF tissues of rats were isolated and cut into approximately 0.5 mm^3^ granules, added to PBS and homogenized by a tissue homogenizer until there were no visible solids. After centrifugation at 3000 RPM for 20 min at 4 °C, the supernatant was collected and used to detect the local concentrations of procollagen type I N-terminal propeptide (PINP) and procollagen type III N-terminal propeptide (PIIINP). The peripheral blood derived from rats was left at room temperature for one hour and then centrifuged at 3000 RPM for 20 min at 4 °C. Blood serum was collected and used to detect the systemic concentrations of PINP and PIIINP. The concentrations of PINP and PIIINP in LF cell supernatant, rat LF and rat blood serum were determined by ELISA kits (Signalway Antibody LLC, Maryland, USA).

### Histological staining and immunohistochemistry analysis

The LF tissues from humans or rats were fixed with 4% paraformaldehyde and then transferred to 10% EDTA for decalcification. Subsequently, the LF tissues were dehydrated, paraffin-embedded, and then cut into 5-μm sections. Histological staining included hematoxylin-eosin (H&E) staining and Elastica-van Gieson (EVG) staining. According to the proportion of elastic fibers and collagen fibers, the LF fibrosis score was assessed as follows: Grade 0 represents normal tissue without a fibrotic region; Grade 1 represents fibrosis involving 0–25% of the entire area; Grade 2 means fibrosis in 25–50% of the LF; Grade 3 indicates between 50 and 75% fibrosis, and Grade 4 indicates fibrosis over 75% of the LF^31^. Antibodies against DCN, TGF-β1, collagen I, collagen III, α-SMA and fibronectin were used for immunohistochemical analysis.

### Statistical analysis

GraphPad Prism V.7.00 software was used for statistical analyses in this study. Continuous data are presented as the mean ± SD, and categorical data are presented as frequencies and percentages. One-way analysis of variance was used for multiple group comparisons, independent t tests were used for the comparison of continuous data between two groups, and chi-square tests were used to analyze the categorical variables. *P* values < 0.05 were considered to be statistically significant.

## Results

### Bioinformatic analysis identifies TGF-β1 as a key regulator in the development of LFH

**Fig. 1:**
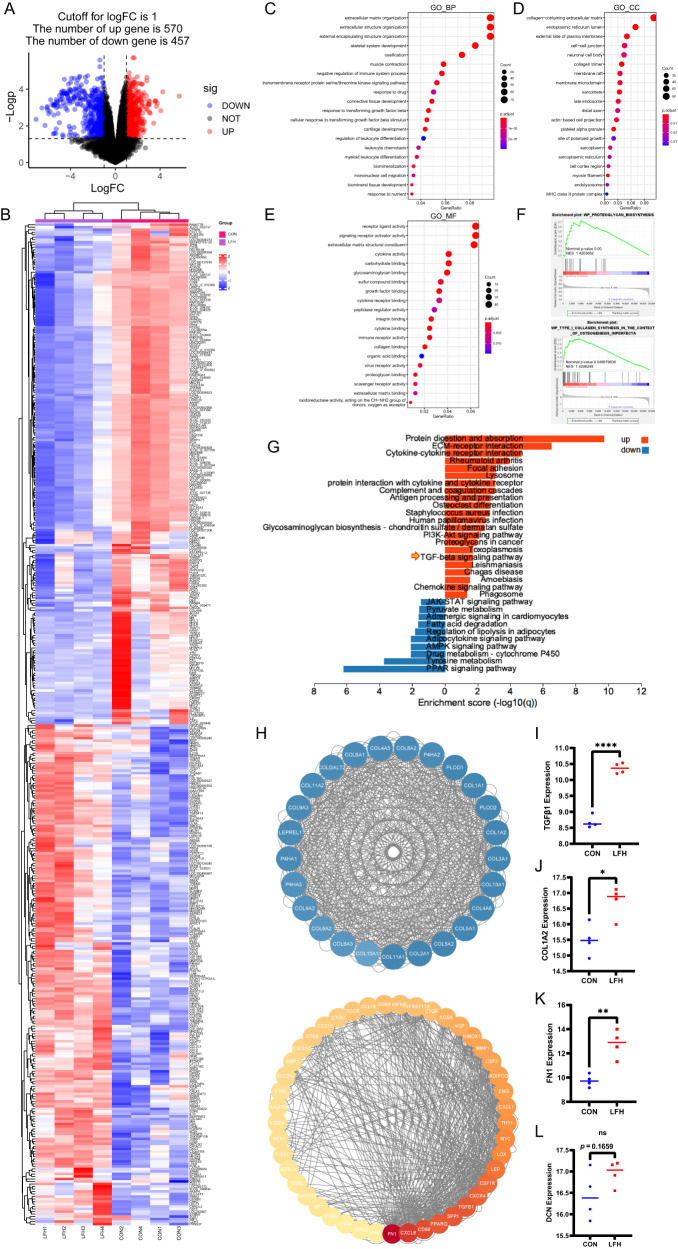
Bioinformatics analysis revealed TGF-β1 as a key regulator in the development of LFH. **A** The volcano plot shows 570 upregulated DEGs and 457 downregulated genes. **B** The heatmap shows the top 200 up- and downregulated genes. **C** Enrichment analysis of biological processes from the GO database. **D** Enrichment analysis of cell components from the GO database. **E** Enrichment analysis of molecular function from the GO database. **F** GSEA of DEGs. **G** KEGG enrichment analysis of the key pathways involved in LFH. **H** PPI analysis identified two major modules. **I**–**L** The relative expression levels of TGF-β1, COL1A2, FN1, and DCN (*n* = 4). Data are presented as the means ± SDs. ns, no significance; *p < 0.05; **p < 0.01; ***p < 0.001.

After QC including PCA and removal of batch effects, we obtained an expression matrix containing 19,410 genes (Supplementary Fig. 1a, b). Differentially expressed genes (DEGs) were further identified with rigorous criteria (logFC >1, *p* < 0.05) (Fig. 1A). The top 200 up- and downregulated genes are represented in Fig. 1B (Supplementary Table 1). GO enrichment analysis of DEGs involving biological process (BP), cell component (CC), and molecular function (MF) was further performed with DEGs, suggesting that DEGs were mainly enriched in terms related to extracellular matrix, such as extracellular matrix organization and collagen-containing extracellular matrix. Interestingly, TGFβ-related biological processes have also been found to play an important role in the pathological process of LFH (Fig. 1C–E). GSEA validated that the process of proteoglycan biosynthesis and type 1 collagen synthesis was significantly enhanced in the progression of LFH (Fig. 1F). Similar results have previously been reported^12,32^. To demonstrate the key pathways involved in LFH, we carried out KEGG analysis, which indicated that the TGFβ pathway was significantly enriched in the pathogenesis of LFH (Fig. 1G). PPI analysis was further performed to explore the key modules in DEGs where two major modules were then identified, in which COL1A2, FN1, and TGFB1, known as TGFβ1, were found to have a relatively higher level of expression (Fig. 1H). These results indicated that COL1A2, FN1, and TGFB1 played an important role in the development of LFH. In addition, we found that these three genes were significantly increased in the samples from LFH patients (Fig. 1I–K). This result showed that the increased expression of TGF-β1 is positively correlated with LFH fibrosis. Our previous work confirmed that DCN played a vital role in the progression of fibrosis by antagonizing TGF-β1^23,25^. For DCN, which encoded protein DCN, we did not observe a significant increase in the LFH samples (Fig. 1L). Therefore, we hypothesized that DCN could be a potential therapeutic candidate for LFH by antagonizing TGF-β1.

### Demographic characteristics of clinical data

**Fig. 2:**
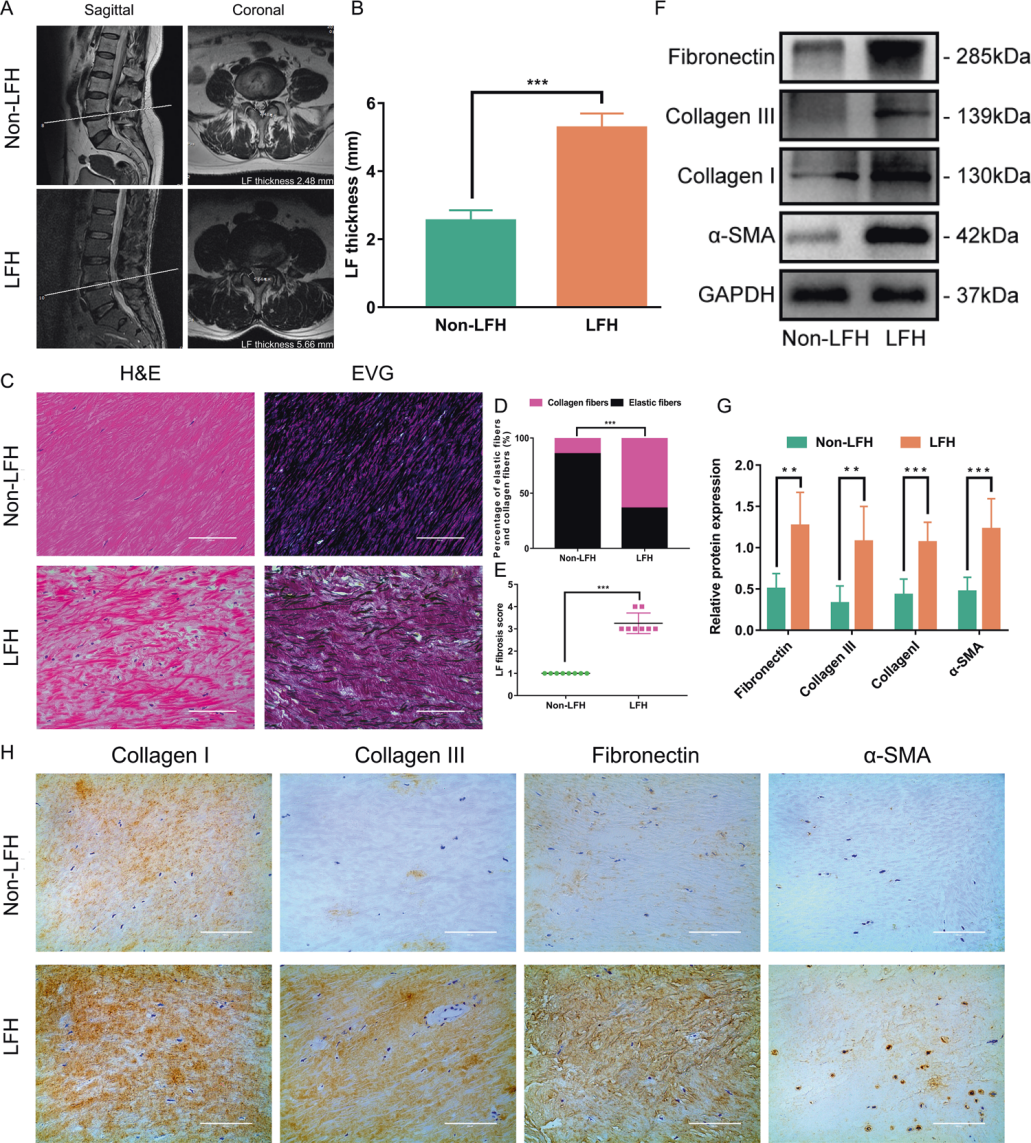
Increased fibrotic degree of LF in patients with LFH. **A** Coronal and sagittal MRI of LF. The LF thickness was determined on coronal MRI. **B** Comparison of LF thickness between the LFH group and the non-LFH group (*n* = 20). **C** Representative images of H&E staining and EVG staining of the LF samples from the two groups (*n* = 8). EVG staining (collagen fibers were stained pink, while elastic fibers were stained black). The scale bar indicates 100 μm. **D** Comparison of the percentage of collagen fibers and elastic fibers between the two groups (*n* = 8). **E** Comparison of LF fibrosis scores between the two groups (*n* = 8). **F** Western blot analysis of collagen I, collagen III, α-SMA and fibronectin protein expression in LF samples from the two groups. GAPDH was the loading control (*n* = 6). **G** Quantitative analysis of collagen I, collagen III, α-SMA and fibronectin protein expression in LF samples from the two groups (*n* = 6). **H** Representative images of immunohistochemical staining of collagen I, collagen III, α-SMA and fibronectin in LF samples from the two groups (*n* = 8). The scale bar indicates 100 μm. Data are presented as the means ± SDs. ns, no significance; *p < 0.05; **p < 0.01; ***p < 0.001.

In view of the results of the mechanistic analysis, we collected some clinical samples for analysis. A total of 40 patients (L4/5) were enrolled in this study: 20 patients in the LFH group and 20 patients in the non-LFH group. Demographic characteristics in both groups are shown in Table 1. Baseline characteristics of sex and bone mass index (BMI) and operative level of the 2 groups were similar. However, the mean age of the LFH group was significantly older than that of the non-LFH group. The mean value for LF thickness in the LFH group was 5.27 ± 0.42 mm, which was significantly higher than that in the non-LFH group (2.54 ± 0.31 mm) (Fig. 2A, B).

**Table 1:** Comparison of the baseline data of patients between the two groups.

Variable	Non-LFH group (*n* = 20)	LFH group (*n* = 20)	*P* value
Age (years)	53.6 ± 8.2	60.2 ± 7.0	0.009
Sex (male/female)	13/7	9/11	0.341
BMI (kg/m^2^)	22.2 ± 2.4	23.6 ± 2.3	0.069

### Increased fibrotic degree of LF in patients with LFH

Many studies have shown that fibrosis is the main pathological course of LFH^4,11,14,32^. Our study emphasized this again. H&E staining showed that the fibrous structure of LF in the LFH group was disordered and uneven. There was a loss of fiber in some areas, and the number of cells was increased (Fig. 2C). Furthermore, the proportion of elastic fibers decreased, and the proportion of collagen fibers increased in EVG staining (Fig. 2C, D). The fibrosis score of the LF in the LFH group was significantly higher than that in the non-LFH group (Fig. 2E). Western blotting analysis and immunohistochemistry analysis showed that the expression of collagen I and collagen III was significantly upregulated in the LHF group (Fig. 2F–H). In addition, we observed increased expression of α-SMA and fibronectin in the LHF group (Fig. 2F–H).

### TGF-β1 and DCN expression was upregulated in LFH

**Fig. 3:**
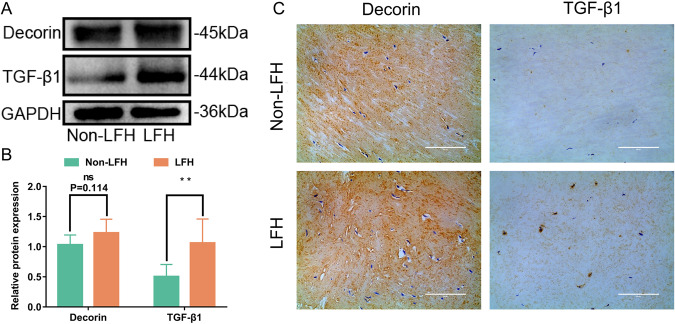
TGF-β1 and DCN expression were upregulated in LFH. **A** Western blot analysis of DCN and TGF-β1 protein expression in LF samples from the two groups. GAPDH was the loading control (*n* = 6). **B** Quantitative analysis of DCN and TGF-β1 protein expression in LF samples from the two groups (*n* = 6). **C** Representative images of immunohistochemical staining of DCN and TGF-β1 in LF samples from the two groups (*n* = 8). The scale bar indicates 100 μm. Data are presented as the means ± SDs. ns, no significance; *p < 0.05; **p < 0.01; ***p < 0.001.

We then examined the expression of TGF-β1 and DCN in LF specimens. Protein analysis and immunohistochemistry analysis showed that the expression of TGF-β1 was significantly increased in the LFH group. For DCN, we observed increased expression in the LFH group, but there was no significant difference (*P* = 0.114) (Fig. 3). Our results were consistent with bioinformatic analysis.

### DCN inhibited LF cell proliferation

**Fig. 4:**
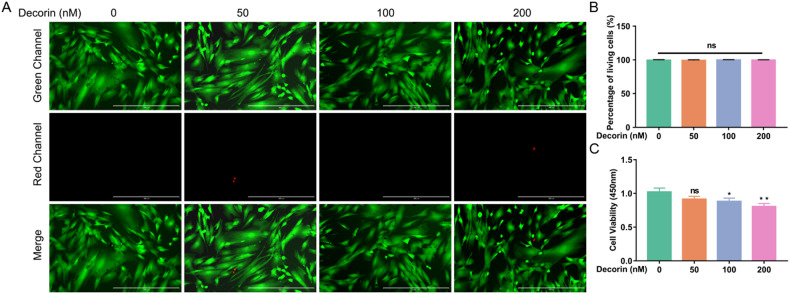
DCN inhibited LF cell proliferation. **A** Calcein-AM/PI double staining of LF cells after intervention with different concentrations of DCN (green, living cells; red, nuclei of dead cells). The scale bar indicates 400 μm. **B** Quantitative analysis of the percentage of living cells (six random fields of view for each well). **C** CCK-8 assay of LF cells showed that DCN suppressed the proliferation of LF cells, especially when the concentration of DCN was 100 nM and 200 nM (*n* = 6). Data are presented as the means ± SDs. *ns* no significance; *p < 0.05; **p < 0.01; ***p < 0.001.

To further investigate the role of DCN in LFH, we isolated human LF cells from LF tissues. First, we detected the effect of DCN on the viability and proliferation of LF cells. The CCK-8 assay showed that DCN suppressed LF cell proliferation, and this inhibitory effect was dose-dependent. LF cell proliferation was obviously inhibited when the concentration of DCN was 100 nM and 200 nM (Fig. 4C). However, with increasing DCN concentration, there was no significant difference in the proportion of living cells and dead cells, which suggested that the inhibitory effect of DCN on LF cell proliferation was not caused by inducing cell death (Fig. 4A, B).

### TGF-β1 induced fibrosis in normal LF cells, while DCN inhibited fibrosis in hypertrophic LF cells

**Fig. 5:**
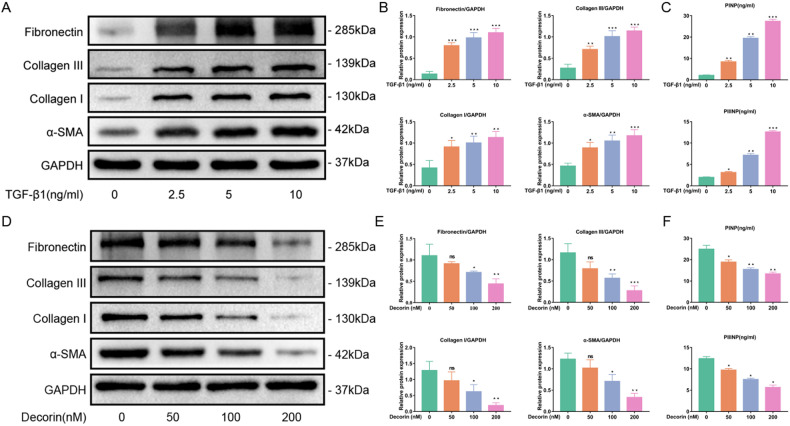
TGF-β1 induced fibrosis in normal LF cells, while DCN inhibited fibrosis in hypertrophic LF cells. **A** Western blot analysis and **B** quantitative analysis of collagen I, collagen III, α-SMA and fibronectin protein expression in normal LF cells after administration of different concentrations of TGF-β1. GAPDH was the loading control (*n* = 3). **C** ELISAs of PINP and PIIINP levels in the cell supernatant of normal LF cells after the administration of different concentrations of TGF-β1 (*n* = 3). **D** Western blot analysis and **E** quantitative analysis of collagen I, collagen III, α-SMA and fibronectin protein expression in hypertrophic LF cells after administration of different concentrations of DCN. GAPDH was the loading control (*n* = 3). **F** ELISAs of PINP and PIIINP levels in the cell supernatant of hypertrophic LF cells after the administration of different concentrations of DCN (*n* = 3). Data are presented as the means ± SDs and compared with those of the control group. ns, no significance; *p < 0.05; **p < 0.01; ***p < 0.001.

Previous literature has demonstrated that TGF-β1 can induce fibrosis in LF cells^11^. This result was also confirmed in our study. After the LF cells were seeded on six-well plates, they were administered with different concentrations of TGF-β1. The results showed that the expression of collagen I, collagen III, α-SMA and fibronectin was significantly increased in ligamentum flavum cells (Fig. 5A, B). The levels of PINP and PIIINP in the cell supernatant were significantly increased with increasing TGF-β1 concentration (Fig. 5C). In addition, we found that DCN inhibited the fibrosis of hypertrophic LF cells. With the administration of DCN, the expression of collagen I, collagen III, α-SMA and fibronectin was significantly downregulated in hypertrophic LF cells, as well as the levels of PINP and PIIINP in the cell supernatant (Fig. 5D–F). These results indicated that TGF-β1 and DCN play opposite roles in LF cell fibrosis. The concentration with the most obvious effect for TGF-β1 (10 µg/ml) and DCN (100 nM and 200 nM) was identified and used for subsequent studies.

### DCN inhibited TGF-β1-induced fibrosis-associated protein expression in LF cells

**Fig. 6:**
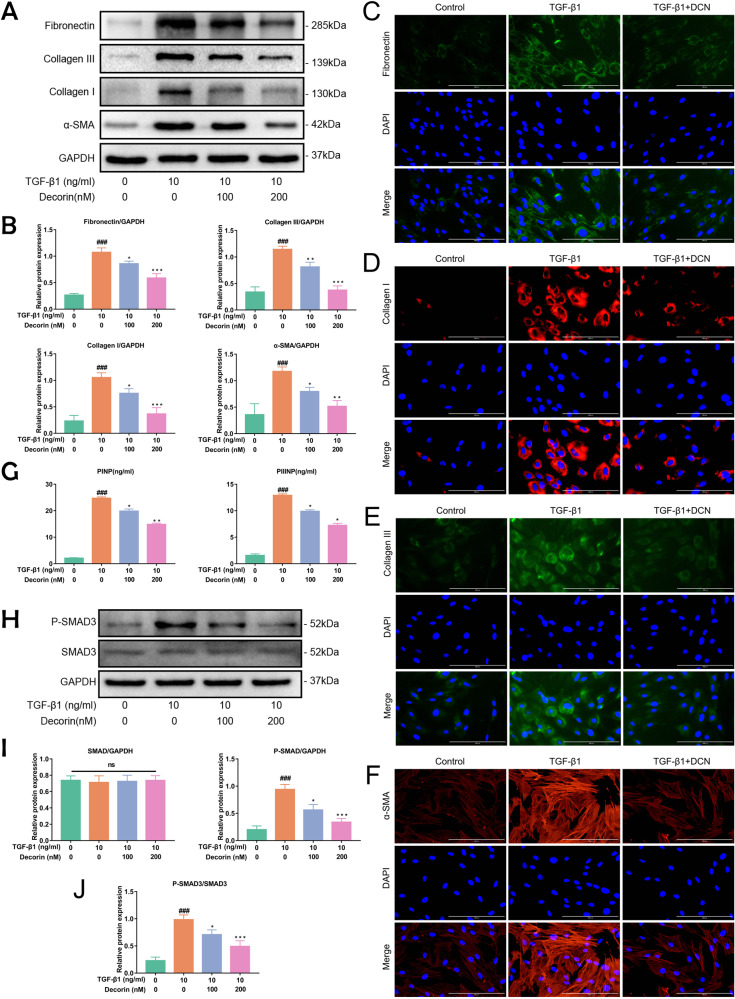
DCN inhibited TGF-β1-induced fibrosis-associated protein expression in LF cells. **A** Western blot analysis and **B** quantitative analysis of the protein expression levels of collagen I, collagen III, α-SMA and fibronectin in LF cells after intervention with 10 ng/ml TGF-β1 and different concentrations of DCN for 24 h (*n* = 3). **C**–**F** Immunofluorescence staining of collagen I, collagen III, α-SMA and fibronectin under different interventions for 24 h (fibronectin and collagen III were stained green; collagen I and α-SMA were stained red; DAPI, blue). Scale bar: 400 µm. **G** The levels of PINP and PIIINP in the cell supernatant of LF cells under different interventions for 24 h (*n* = 3). **H** Western blot analysis and **I**–**J** quantitative analysis of SMAD3 signaling protein expression levels in LF cells after intervention with 10 ng/ml TGF-β1 and different concentrations of DCN for 24 h (*n* = 3). Data are presented as the means ± SDs. ###*p* < 0.001 vs. the control group; *p < 0.05 vs. the TGF-β1 group; **p < 0.01 vs. the TGF-β1 group; ***p < 0.001 vs. the TGF-β1 group.

We investigated the interaction of DCN and TGF-β1 in LF cells. Normal LF cells were administered 10 ng/ml TGF-β1 in combination with different concentrations of DCN (0 nM, 100 nM, and 200 nM). The results showed that the expression of collagen I, collagen III, α-SMA, and fibronectin was significantly increased with 10 ng/ml TGF-β1, yet this phenomenon was alleviated after DCN intervention (Fig. 6A, B). These results were verified by immunofluorescence (Fig. 6C–F). Consistent with the protein expression of fibrotic proteins, the increased levels of PINP and PIIINP induced by TGF-β1 were significantly attenuated by DCN in the cell supernatant (Fig. 6G). Our data showed that DCN can inhibit the fibrosis of LF cells by antagonizing TGF-β1. In addition, further analysis was performed to investigate the ability of DCN to inhibit TGF-β1-stimulated fibrotic protein expression in LF cells through the SMAD3 signaling pathway. Western blotting analysis revealed that intervention with DCN in LF cells inhibited TGF-β1-stimulated P-SMAD3 protein expression (Fig. 6H–J). In summary, our data indicated that DCN suppressed TGF-β1-induced fibrosis of LF cells by blocking the SMAD3 signaling pathway.

### DCN alleviated LF fibrosis and hypertrophy in an in vivo LFH model

**Fig. 7:**
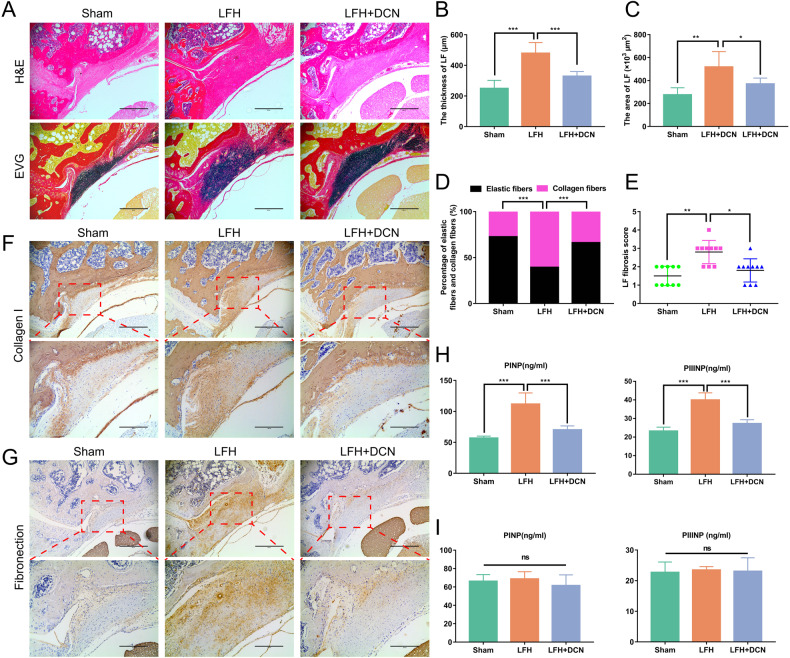
DCN ameliorates mechanical stress-induced LFH in vivo. **A** Representative images of H&E- and EVG-stained LF tissues from rats in each group. Scale bar: 400 µm. **B**–**E** Quantitative analysis of the LF thickness, LF area, percentage of collagen fibers and elastic fibers, and LF fibrosis score in H&E- and EVG-stained sections (*n* = 10 rats in each group). **F**, **G** Representative images of immunohistochemical staining for collagen I and fibronectin in rat LF tissues under different interventions after 8 weeks. Scale bar: 400 µm (lower power lens); 200 µm (high power lens). **H** ELISAs of PINP and PIIINP levels in LF tissues from rats (*n* = 10 rats in each group). **I** ELISAs of PINP and PIIINP levels in serum samples from rats (*n* = 10 rats in each group). Data are presented as the means ± SDs. ns, no significance; *p < 0.05; **p < 0.01; ***p < 0.001.

We further explored the effect of DCN in an LFH model of lumbar instability induced by resection of the posterior structure of the lumbar spine. Compared with those of the sham group, the thickness and area of LF in the LFH model group were significantly increased. Histological analysis showed that the morphological changes in LF in the LFH model group were consistent with those in human LF isolated from the LFH group, including disordered, uneven, focal loss of fiber structure, a decreased proportion of elastic fibers and an increased proportion of collagen fibers. However, in the DCN treatment group, we found that those morphological changes in the LFH model group were significantly ameliorated (Fig. 7A–E). In addition, immunohistochemistry analysis showed that the expression levels of collagen I and fibronectin in the LFH model groups were significantly higher than those in the sham group, yet this phenomenon was significantly ameliorated after DCN intervention (Fig. 7F, G). Similarly, ELISAs showed that the local levels of PINP and PIIINP in the LFH model group were significantly increased compared to those in the sham group. However, in the DCN treatment group, this phenomenon was significantly ameliorated (Fig. 7H). To further test the systemic effects of local DCN intervention, we detected the levels of PINP and PIIINP in peripheral blood by ELISA. The results showed no significant difference in serum concentrations of PINP and PIIINP from peripheral blood among the three groups, which indicated that the application of local DCN will not cause systemic effects (Fig. 7I).

## Discussion

LFH is considered to play a pivotal role in the pathogenesis of LSCS^2–4^. The development of LFH is affected by many factors, including mechanical stress, age, sex, obesity and diabetes mellitus; however, the specific mechanism has not yet been completely clarified^11,14,33,34^. A variety of studies have shown that the development of LFH is closely related to fibrosis, and multiple cell factors participate in this process, among which TGF-β1 is known to be crucial in the development of LFH pathology^4,7,11,13–18,35,36^. In the present study, the bioinformatic analysis and in vivo and in vitro experiments again emphasize this finding.

The role of TGF‐β1 has been investigated in various fibrosis-associated pathological processes, including lung and kidney fibrosis, joint contracture, scar repair, liver cirrhosis, postoperative epidural adhesions, and atherosclerosis^22,23,25,35,37–39^. TGF‐β1 can activate the TGF‐β1/Smad3 pathway, thereby regulating the differentiation from fibroblasts to myofibroblasts though the upregulation of α-SMA^23,35,36^. In addition, TGF-β1 can induce the synthesis of multiple ECM components, including collagen I, collagen III, and fibronectin^23,36^. As one type of fibroblast, the differentiation of LF cells is also regulated by TGF‐β1. In our study, we also found that the expression of collagen I, collagen III, α-SMA, and fibronectin was increased in hypertrophic LF tissues and coincided with TGF‐β1 upregulation, and these findings were confirmed again by in vitro experiments.

Given the crucial role of TGF‐β1 in the development of LFH, inhibiting the TGF‐β1 pathway is a potential therapeutic option to treat LFH or ameliorate its severity. Although several studies have found that many proteins, including CCN5/WISP‐2, clusterin, cytokine receptor-like factor 1 (CRLF1) and epidermal growth factor (EGF), can regulate the development of LFH via the TGF‐β1 pathway, there is still a lack of effective targeted drugs and nonsurgical treatments to prevent the development of LFH^35,36,40,41^.

As one of the components of the extracellular matrix, DCN showed significant antifibrotic effects on multiple fibrosis-associated disease models in the kidney, liver, lung, optic nerve and vasculature by inhibiting the TGF‐β1 pathway^24,38,39,42–44^. It has been reported that DCN can reduce tissue fibrosis by binding TGF‐β1 and neutralizing part of its activities and is considered a natural inhibitor of TGF‐β1^21^. Mechanistic studies indicated that DCN inhibits the TGF‐β1 pathway by blocking the TGF-β1-induced phosphorylation of SMAD2/3, thereby downregulating the expression of collagen I, collagen III, α-SMA and fibronectin^23,39^. Therefore, we propose a hypothesis that DCN can be a potential therapeutic candidate for LFH.

The relationship between DCN and TGF‐β1 is rather complex. On the one hand, TGF‐β1 can induce increased expression of DCN^45^. On the other hand, DCN can inhibit TGF‐β1-induced fibrosis^46^. Previous literature has shown that the expression of DCN is increased in fibrotic tissue^38,39,47^. In addition, Yabe et al. reported that the expression of DCN was significantly increased in LFH^4,32^. These studies suggested that DCN has a protective role in LFH. In our study, bioinformatic analysis and protein analysis of LF tissues showed that the expression of DCN in LFH was increased, but there was no significant difference. We think the reasons may be as follows. First, in the early stage of LFH, the increased expression of TGF‐β1 induced by mechanical stretching force upregulated the expression of DCN. At this stage, DCN expression increased significantly in LFH^4,11,30,32^. Increased TGF‐β1 activates the TGF‐β1/SMAD3 signaling pathway, leading to fibrosis and hypertrophy of the LF. With the development of LFH, part of the DCN participates in the repair of hypertrophic LF, and part of the DCN binds TGF‐β1 to suppress TGF-β1-induced fibrosis^48,49^. As a result, the increase in DCN expression cannot match the increase in TGF expression, thus further aggravating the development of LFH.

Based on the above results, we assume that exogenous DCN can help alleviate LFH by antagonizing TGF‐β1, and this hypothesis was confirmed by in vitro cell experiments. With the administration of DCN, fibrosis-associated protein expression in hypertrophic LF cells was significantly downregulated. In addition, our study indicated that TGF‐β1 upregulated the expression of fibrosis-associated protein in LF cells, and this induced effect was inhibited after the administration of DCN by blocking the TGF‐β1/SMAD3 signaling pathway. These findings are important experimental evidence that DCN can inhibit LF fibrosis by antagonizing TGF-β1, indicating that DCN is a potential therapeutic candidate for LFH.

To further investigate the anti-hypertrophic effects of DCN on LF in vivo, we built an LFH rat model as described in previous literature^30^. Increased mechanical stress is the main cause of LFH^11,14,30^. Damage to the posterior structural integrity of the lumbar spine can cause segmental instability, thereby producing increased mechanical stress on the LF, ultimately leading to LFH^11,30,31^. It was also found that the expression of TGF-β1 was significantly increased in LF cells subjected to mechanical stretching force, and the application of exogenous TGF-β1 can induce the synthesis of collagen in LF cells^11^. These studies indicated that TGF-β1 plays a crucial role in LFH induced by mechanical stress. In the present study, we found that the LF thickness was significantly increased in the LFH model group and coincided with the increased fibrosis score and upregulation of collagen I and fibronectin. In contrast, in the DCN treatment group, the thickness of LF was thinner than that in the LFH model group, and the expression of collagen I and fibronectin was also decreased.

In addition, the local levels of PINP and PIIINP were significantly increased in the LFH model group. However, in the DCN treatment group, the local levels of PINP and PIIINP were significantly lower than those in the LFH model group. While excessive fibrosis can lead to the development of multiple fibrosis-related diseases, moderate fibrosis is also key to wound healing and tissue repair^50–52^. Given the extensive antifibrotic effect of DCN, we detected the serum levels of PINP and PIIINP to investigate whether topical application of DCN caused systemic effects. The results showed that the serum levels of PINP and PIIINP in peripheral blood among the three groups were similar. We believe that the reason for the difference between local and systemic levels of PINP and PIIINP may be the method of DCN administration. Since the blood supply around LF is poor, the local application of DCN cannot effectively reach other tissues and organs through blood circulation and therefore does not cause systemic effects. In summary, our data indicated that local application of DCN can ameliorate mechanical stress-induced LFH by antagonizing TGF-β1 in vivo without causing systemic effects.

Nevertheless, limitations still existed in this study. First, we showed that DCN can ameliorate mechanical stress-induced LFH by antagonizing TGF-β1 in vivo, yet the most effective concentration of DCN remains unclear. Although our data suggested that the effect of DCN administered by paravertebral injections is limited to the area of the LF and did not affect the serum levels of PINP and PIIINP, further studies are needed to assess the effective concentration range and safety. Moreover, a noninvasive and more effective drug delivery method targeting LF should be explored and developed.

In conclusion, our research showed that DCN can ameliorate the development of LFH by antagonizing TGF-β1, which indicates that DCN is a potential therapeutic candidate for LSCS caused by LFH.

## Availability of data and materials

The datasets used and/or analyzed during the current study are available from the corresponding author upon reasonable request.

## Supplementary information

Supplementary information

supplementary table1
