# Supplementary material for: Decorin: a potential therapeutic candidate for ligamentum flavum hypertrophy by antagonizing TGF-β1
Source: Exp Mol Med. 2023 Jul 3;55(7):1413–23. doi: 10.1038/s12276-023-01023-y (PMC10394053; doi:10.1038/s12276-023-01023-y)
Supplement: Supplementary file 1 — Supplementary information [file 12276_2023_1023_MOESM1_ESM.pdf]

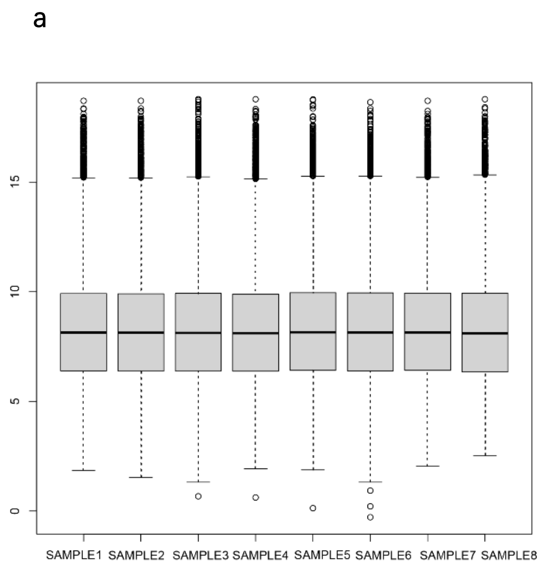

Supplementary Fig. 1. (a) Quality control of the raw data. Boxplot represented that the batch effect had been removed. (b) Samples from normal people or LFH patients could be distinguished according to PCA analysis.
